# Analysis of Transcripts in the Fly Cell Atlas Reveals Additional Cell Populations in the *Drosophila melanogaster* Ovary

**DOI:** 10.17912/micropub.biology.001725

**Published:** 2025-07-10

**Authors:** Oscar Mendoza Andrade, Zach Wright, Sahel Ghasemzadeh, Dan T Bergstralh

**Affiliations:** 1 Division of Biological Sciences, University of Missouri, Columbia, MO, 65201, USA.

## Abstract

The *Drosophila* ovary serves as a powerful model system for epithelial morphogenesis. In this study we analyzed previously unidentified ovarian epithelial cells from the Fly Cell Atlas dataset. We identified eight transcriptionally distinct clusters and annotated six of them, including follicle cell developmental stages 9, 10A, and 10B/11. Two additional clusters remain only weakly identified. This work facilitates future use of the ovarian Fly Cell Atlas by providing validated developmental stage markers and filling critical gaps in follicle cell annotation.

**Figure 1. Annotation of previously unidentified cell populations in the Fly Cell Atlas of the Drosophila ovary:**
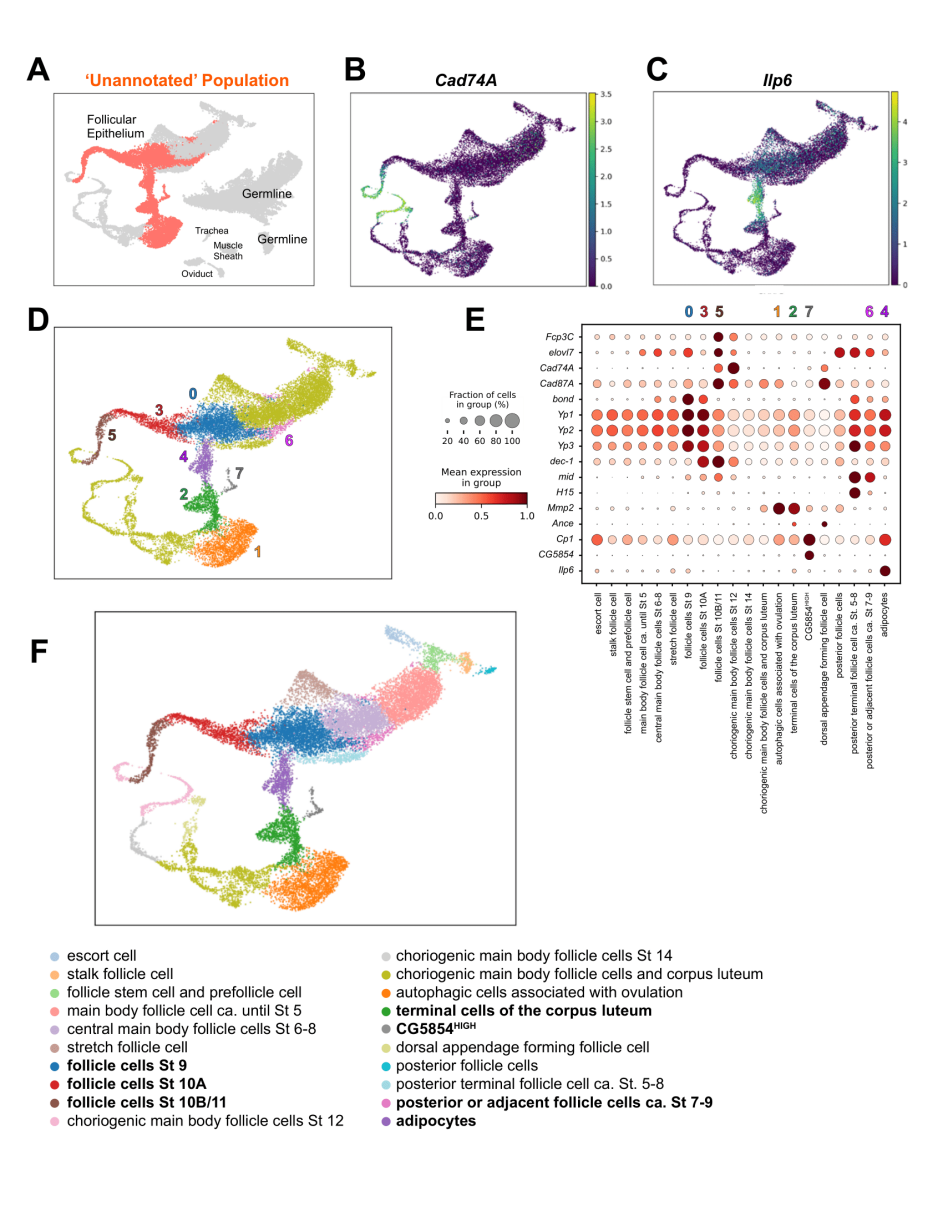
A) Highlighted is the size and position of the “unannotated” population in the ovarian epithelial Fly Cell Atlas UMAP. B and C) Exploratory analysis of two marker genes, Cad74A (B) and Ilp6 (C). mRNA expression is indicated by color, ranging from low (blue) to high (yellow). D) Projection of eight (coded 0-7) transcriptionally distinct cluster assignments for unannotated cells onto the UMAP. E) A dot plot graph shows expression patterns of key marker genes across all annotated follicle cell clusters. The size of each dot represents the percentage of cells within a cluster expressing a given gene, while the color scale indicates average expression level. F) UMAP visualization displaying annotated cell populations, integrating new cellular identities defined by marker genes identified in this study.

## Description

The *Drosophila* ovary is a well-established model system for the study of epithelial morphogenesis (Duhart et al., 2017; Horne-Badovinac and Bilder 2005). The ovary is divided into ovarioles, which are strings of individual egg chambers at increasing stages of maturity. By convention, these stages are numbered 1-14. Each egg chamber is surrounded by an epithelial tissue, the follicular epithelium, that undergoes dramatic morphological changes over the course of egg chamber maturation. Multiple labs have used single-cell RNA Sequencing technology to identify and characterize follicle cell transcriptomes as these changes occur. A number of these and other *Drosophila* RNA-Seq studies have been collected and collated to provide a readily-navigated and publicly-available transcriptomic map, The Fly Cell Atlas (FCA) (Li et al., 2022). The FCA is a powerful resource for the cell and developmental biology communities, particularly those scientists working with *Drosophila.* As of June 2025, the FCA of the adult *Drosophila* ovary identifies 34 distinct transcriptional clusters encompassing both germline and somatic cell types. Notably missing from the current set of follicle cell annotations are developmental Stages 9, 10A, 10B, and 11, which represent an important developmental window that includes patency and the development of septate junctions (Isasti-Sanchez et al., 2021; Rice et al., 2021; Row et al., 2021). Concomitantly, a large population in the epithelial cell space of the FCA UMAP (Uniform Manifold Approximation and Projection) is labelled as “unannotated” ( Figure 1A ), indicating these cells could not be confidently assigned to known lineages due to absent canonical markers, poor transcriptional alignment, or technical artifacts (Li et al., 2022). On the UMAP, the “unannotated” population is bordered by the following annotated populations: stretch follicle cells; central main body follicle cells St 6-8; posterior follicle cells ca. St 6-8; choriogenic main body follicle cells St 12; and choriogenic main body follicle cells and corpus luteum. This suggests that the unannotated population includes follicle epithelial cells at Stages 9, 10A, 10B, and 11.

To investigate further, we examined two reference markers from earlier ovarian RNA-seq analysis (Jevitt et al., 2020): *Cad74A* (follicular cells stages 10B-12) (Zartman et al., 2009) and *Ilp6* (adipocytes) (Okamoto et al., 2009; Slaidina et al., 2009). We found that they demarcated two distinct subpopulations within the unannotated population (Figures 1B and 1C). At resolution 0.1, *Cad74A ^+^* or *Ilp6 ^+^* cells clustered broadly with marker-negative cells. At resolution 0.3, these populations separated. Resolution 0.2 yielded eight distinct clusters (initially numbered 0-7), including one defined by *Cad74A* and another by *Ilp6* ( Figure 1D ). We then applied a Wilcoxon rank-sum test ('one cluster versus the rest') to identify the most discriminatory marker genes for each cluster. Extended Data Table 1 provides these gene lists for the eight new clusters.

**Clusters 0, 3, and 5:** We identify these as mid-stage (9-11) follicle cells based on 1) the topographical position of these clusters on the UMAP, which is consistent with a developmental trajectory that extends from follicle stem cells and prefollicle cells to the choriogenic follicle cell populations and 2) the expression of previously reported markers ( Figure 1E ).

*Fcp3C* and *elovl7* mark Stages 10B/11 (Tootle et al., 2011), and *Cad74A* and *Cad87A* are upregulated in these stages (Zartman et al., 2009). We therefore identify Cluster 5 as “follicle cells Stages 10B/11.” *bond* is most highly expressed in Stages 9 and 10A (Szafer-Glusman et al., 2008), as are the yolk protein genes *Yp1, Yp2, and Yp3* (Tootle et al., 2011). These markers are expressed in both Clusters 0 and 3, and cannot distinguish between them.

Since egg chamber rotation—driven by extracellular matrix deposition and collective epithelial migration—concludes at Stage 9 (Cetera and Horne-Badovinac 2015), we investigated whether genes involved in these processes show differential expression between Clusters 0 and 3. We generated lists of genes that were significantly more highly expressed in one cluster versus the other. Genes with higher expression in Cluster 0 showed strong association with the Gene Ontology term ‘Epithelial Cell Migration’ (GO:0010631, *p* =5.717×10⁻⁵). Additionally, thirteen genes from this list— *sona* , *trol* , *LanB1* , *vkg* , *Col4a1* , *LanA* , *SPARC* , *dsx-c73A* , *LanB2* , *Fili* , *dlp* , *CG5757* , and *AdamTS-A* —are associated with the GO term ‘Extracellular Matrix’ (GO:0031012). In contrast, genes more highly expressed in Cluster 3 showed no significant association with epithelial cell migration. However, twelve genes from this list are found in GO:0031012: *Vm26Aa* , *Vm34Ca* , *dally* , *CG14309* , *psd* , *mfas* , *Vm32E* , *Vm26Ac* , *tyn* , *Vml* , *frac* , and *ltl* . Notably, five of these genes are associated with the vitelline membrane, and the eggshell gene *nudel* shows high expression in Cluster 0. These findings suggest that Cluster 3 represents a later developmental stage than Cluster 0, consistent with its position on the UMAP.

We therefore identify Cluster 0 as "follicle cells Stage 9" and Cluster 3 as "follicle cells Stage 10." A complication to this identification is that *dec-1* , previously used to mark Stage 9-12 follicle cells (Jevitt et al., 2020), is not expressed in Cluster 3. However, our analysis of a subsequent transcriptomic study (Slaidina et al., 2021) did not reveal *dec-1* at Stage 9 and is consistent with our other markers.

**Cluster 6** : Like the annotated “posterior terminal follicle cell ca. St. 5-8” population, this cluster demonstrates high expression of *midline* ( Figure 1E ). Midline is a T-box transcription factor expressed in posterior follicle cells, with protein evident by Stage 8 (Fregoso Lomas et al., 2013). Another T-box transcription factor, H15, is also expressed in posterior follicle cells, but by Stage 10A, H15 protein is obviously restricted to a smaller posterior region than Midline (Fregoso Lomas et al., 2013). *H15* expression is not evident in Cluster 6. Similar cells were identified in another transcriptomic dataset as posterior terminal follicle cells at stages 7-9 (Slaidina et al., 2021). We therefore identify Cluster 6 as “posterior or adjacent follicle cells ca. St 7-9.”

**Clusters 1 and 2** : Both of these clusters are marked by expression of *Mmp2* , which encodes Matrix metalloprotease 2. Mmp2 is required for follicle trimming (the degradation of posterior follicle cells surrounding a mature oocyte during ovulation) and corpus luteum formation (Deady et al., 2015). Mmp2::GFP is observed in follicle cells at the posterior and a subset of anterior cells in Stage 14 egg chambers and also at the anterior and posterior of the corpus luteum (Deady et al., 2015). Unlike Cluster 1, Cluster 2 contains cells expressing *Ance* ( Figure 1E ), which is expressed in a subset of cells at the termini of the corpus luteum (Jevitt et al., 2020) and in the dorsal appendage forming cells. Cluster 2 is also distinguished from Cluster 1 by some expression of *Atf3* and by stronger expression of *diap1* . Two populations determined in a previous study share these profiles (Jevitt et al., 2020) and were both identified as “terminal cells of the corpus luteum.”

We are confident in the identification of Cluster 2, and have named this cluster accordingly, but we are less sure of the identity of Cluster 1. To help distinguish between them, we generated lists of genes more significantly expressed in one than the other and performed Gene Ontology analysis. Unlike Cluster 2, the list of genes more highly expressed in Cluster 1 is significantly associated with the GO Terms ‘Programmed Cell Death Involved in Cell Development’ (GO:0010623, *p=* 1.980×10 ^-7^ ) and ‘Autophagy’ (GO:0006914, *p=* 2.337×10 ^-7^ ): associated genes include *dcp-1, Atg1, Atg7, Atg8A, Atg9, Atg17,* and *Atg18A* . These findings suggest the possibility that the cells in Cluster 1 are involved in follicle trimming, but without further validation we identify Cluster 1 as “autophagic cells associated with ovulation.”

**Cluster 7:** This cluster is marked by *Cp1, Mmp1, Sap-R, GLaz* and several uncharacterized genes - *CG5854, CG14764, CG5446, CG3348.* We investigated other ovarian transcriptomic datasets (Jevitt et al., 2020; Miao et al., 2024; Slaidina et al., 2021) and found that a similar profile has been previously identified as “Stretched Cells 3,” one of three stretch follicle cell populations (Jevitt et al., 2020). *Cp1* and *Mmp1* encode genes directly implicated in phagocytosis (Purice et al., 2017; Xu et al., 2020), and *Sap-R* and *GLaz* encode genes involved in lysosomal function (Pascua-Maestro et al., 2017; Sellin et al., 2017). Stretch follicle cells also perform phagocytosis and the similarity between “Stretched Cells 3” and Cluster 7 makes it tempting to speculate that both represent phagocytic cells. Based on its similarity to terminal cells of the corpus luteum, Cluster 7 would likely be involved in clearing debris at/after ovulation. However, this possibility is untested and we therefore identify this population as “CG5854 ^HIGH^ ” ( Figure 1E ).

**Cluster 4** : Adipocytes were identified in another ovarian transcriptomics dataset (Jevitt et al., 2020), presumably due to technical difficulty in fully separating the ovary from the fat body during dissection. Cluster 4 has the highest expression of *Ilp6* ( Figure 1E ) and is therefore identified as adipocytes, in agreement with that work.

These annotations fill critical gaps in the Fly Cell Atlas and provide validated markers for future studies of follicle cell development and ovarian morphogenesis.

## Methods

The Drosophila melanogaster ovarian follicular cell population was analyzed using single cell RNA-seq sequencing data available in the Fly Cell Atlas repository at https://flycellatlas.org/. For bioinformatics processing, the Python language was used together with the specialized Scanpy library. To focus specifically on follicular cells, we manually delineated the follicle cell region based on the original UMAP projection provided by the authors and selected the corresponding subset of cells for downstream analysis. A small subpopulation of the “unannotated” cells (119 of 8825, or ~1.4%) falls in the germline cell space on the UMAP, meaning that these are not likely to be somatic. We excluded these cells from consideration. Additional somatic cell annotations – oviduct, ovarian sheath muscle, and adult trachea cells - are also not considered in this study.

An unsupervised clustering analysis was performed on the previously unannotated population using the Leiden algorithm with a resolution of 0.2. This clustering approach was adopted to maintain methodological consistency with the analysis used in the Fly Cell Atlas, facilitating reproducibility and comparability of results. To characterize each cluster, specific marker genes were identified using Scanpy's (version 1.13.1) sc.get_rank_genes_groups_df() function, employing the Wilcoxon rank-sum test to detect significantly differentially expressed genes. Marker genes were filtered using a threshold of p-value <0.05 and a positive z-score (Scanpy's "scores"). Fold change was calculated as the ratio of average counts between the target cluster and the rest. Functional enrichment analyses were conducted using ShinyGO (version 0.82) (Ge et al., 2020) and g:Profiler (Kolberg et al., 2023) to identify overrepresented Gene Ontology biological processes within each cluster.

Our study also made use of three single-cell RNA sequencing datasets derived from *Drosophila* ovaries. Two of these datasets were retrieved from the NCBI Gene Expression Omnibus: GSE162192 (Slaidina et al., 2021), and GSE146040 (Jevitt et al., 2020). We obtained the third dataset (Miao et al., 2024) directly from the authors, but it is also available through NCBI BioProjects (Accession: PRJNA1108780). All datasets were processed and analyzed using the Seurat package (version 5.2) within the RStudio environment.

Generative AI was used to help with language translation (Spanish to English) and copy-editing in preparing this manuscript.

## Data Availability

Description: Gene lists used to identify the eight newly identified clusters of previously ‘unannotated follicular’ cells. Genes in each list demonstrate significantly higher expression in that cluster compared to all other cells in the group examined in this study (p < 0.05). The genes are ranked according to their Wilcoxon z-score, which reflects the strength and consistency of differential expression across cells in the cluster. Higher values indicate stronger upregulation relative to the rest of the dataset. Also shown are the fold change in average gene expression between the cluster and all other cells, the p-value from the Wilcoxon rank-sum test (p value), and the adjusted p-value controlling for multiple comparisons using the Benjamini–Hochberg procedure (adjusted pval). Mean expression values are reported as UMI (Unique Molecular Identifier) counts within the cluster (mean expression in cluster) and in the remaining cells (mean expression outside the cluster).. Resource Type: Dataset. DOI: https://doi.org/10.22002/fjt12-sdx74
